# The Prone Position for the Administration of Anæsthetics

**Published:** 1883-11

**Authors:** 


### THE PRONE POSITION FOR THE ADMINISTRATION OF ANÆSTHETICS.

It is well known that an erect position of the patient adds materially to the dangers attending the administration of chloroform, ether, etc., but it has been thought a necessity in dental practice. Ordinary operating chairs are not intended for a recumbent attitude, and very few of the practicing dentists who use anæsthetics largely are provided with any other convenience. Yet any one is criminally negligent who does not secure every safeguard against danger in such a grave matter as the administration of an anæsthetic.

The erect position is not only dangerous because of obstructions to the free flow of the blood column, but it adds the peril of the lodgment of foreign bodies in the trachea. In the extraction of teeth they are apt, in the hurry of the operation, to slip from the forceps, and are liable to drop into the pharynx, whence their dislodgement is sometimes difficult. Pieces of broken teeth, mouth props, and clots of blood occasionally are sources of danger, and instances are on record where they have caused the death of the patient. If this attitude was an absolute necessity there would be a better excuse in case of such an accident. But we have found that a prone position offers many advantages for the performance of oral surgery, while it simplifies the administration of the anæsthetic.

An operating *table* is easily prepared by taking the frame-work of an ordinary narrow cot bedstead, and stretching the canvas tightly over it. A canvas pillow should be provided for use during the administration of the agent, and straps to hold the arms and body are easily attached beneath it, for use if the patient becomes violent. They need not be in sight, or be brought into use unless a necessity for their employment arises. Such a table should be placed before a window in an unused room, or it may even be set in the laboratory. When a patient is to be anæsthetized, place him or her upon it and proceed with the administration. When this is accomplished, remove the pillow and perform the operation. If teeth are to be extracted the head should be turned to one side or the other, as convenience demands, so that the blood and any extracted teeth or broken pieces will fall to the corner of the mouth, whence they are easily removed. If right inferior teeth are to be removed, and the operator is standing upon the right side of the patient, let him quickly turn the head from him until it rests upon the left cheek, then bending across the body the teeth are presented in a very favorable position for removal. If the left superior jaw is to be operated upon, the position of the head of the patient should be reversed. For inferior teeth upon the left side, a convenient position for the operator is at the head of the patient, who is lying upon the left side. In such position the blood flows easily and readily from the mouth, and the strangling and choking so common in patients in a sitting posture will not be observed.

We have used this position for some time, but our attention has been again called to its advisability by the perusal of a pamphlet by Prof. Tiffany, of the University of Maryland, who recommends it in cases of tracheotomy, and the removal of pharyngeal tumors. In the removal of considerable portions of the upper jaw it has sometimes been found necessary to perform tracheotomy on account of the accumulation of blood, but by the use of the prone position this will be unnecessary, as the blood readily flows from the mouth. Prof. Tiffany recommends that the head should be slightly raised. We have found that it is better to allow the head to be in a line with, or even sometimes a little lower than the body, the removal of the blood being effected by turning the head to one side or the other. In operations in dental surgery, performed in this manner, we have never known the blood to cause any embarrassment whatever.

